# Association of Prenatal Maternal Anxiety With Fetal Regional Brain Connectivity

**DOI:** 10.1001/jamanetworkopen.2020.22349

**Published:** 2020-12-07

**Authors:** Josepheen De Asis-Cruz, Dhineshvikram Krishnamurthy, Li Zhao, Kushal Kapse, Gilbert Vezina, Nickie Andescavage, Jessica Quistorff, Catherine Lopez, Catherine Limperopoulos

**Affiliations:** 1 Division of Diagnostic Imaging and Radiology, Children’s National, Washington DC; 2 Division of Neonatology, Children’s National, Washington DC; 3 Department of Pediatrics, The George Washington University School of Medicine, Washington DC

## Abstract

**Importance:**

Maternal psychological distress during pregnancy is associated with adverse obstetric outcomes and neuropsychiatric deficits in children. Currently unavailable in vivo interrogation of fetal brain function could provide critical insights into the onset and timing of altered neurodevelopmental trajectories.

**Objective:**

To investigate the association between prenatal maternal stress, anxiety, and depression and in vivo fetal brain resting state functional connectivity.

**Design, Setting, and Participants:**

This cohort study included pregnant women scanned between January 2016 and April 2019. A total of 50 pregnant women with healthy pregnancies were prospectively recruited from low-risk obstetric clinics in the Washington DC area and were scanned at Children’s National in Washington DC.

**Exposures:**

Maternal stress, anxiety, and depression.

**Main Outcomes and Measures:**

The association of prenatal maternal stress, anxiety, and depression with whole-brain connectivity was analyzed using multivariate distance matrix regression. Prenatal maternal stress, anxiety, and depression were assessed using the Perceived Stress Scale, Spielberger State Anxiety Inventory and Spielberger Trait Anxiety Inventory, and the Edinburgh Postnatal Depression Scale, respectively. Whole-brain connectivity was measured from 100 functionally defined regions of interest.

**Results:**

This study analyzed 59 resting-state functional connectivity magnetic resonance image data sets from the fetuses (mean [SD] gestational age, 33.52 [4 weeks]) of 50 healthy pregnant women (mean [SD] age, 33.77 [5.51]). Mean (SD) scores for the questionnaires were as follows: Spielberger State Anxiety Inventory, 26.66 (6.72) (range, 20-48); Spielberger Trait Anxiety Inventory, 28.09 (6.62) (range, 20-50); Perceived Stress Scale, 9.27 (5.13) (range, 1-25); and Edinburgh Postnatal Depression Scale 3.24 (2.84) (range, 0-14). Prenatal maternal anxiety scores measured using the Spielberger Trait and State Anxiety Inventories were associated with differences in fetal connectivity (Spielberger State Anxiety Inventory: pseudo-*R*^2^ = 0.019, *P* = .04; Spielberger Trait Anxiety Inventory: pseudo-*R*^2^ = 0.021, *P* = .007). Interhemispheric connections, such as those involving the parietofrontal and occipital association cortices, were associated with reduced maternal prenatal anxiety, and those between the brainstem and sensorimotor areas were associated with higher anxiety scores.

**Conclusions and Relevance:**

In this cohort study, an association was found between prenatal maternal anxiety and disturbances in fetal brain functional connectivity, suggesting altered fetal programming. Early onset of functional deviations suggests the need for more widespread screening of pregnant women for symptoms of anxiety.

## Key Points

**Question:**

What is the association between maternal psychological distress and fetal brain functional connectivity?

**Findings:**

In this cohort study of 50 women with 59 fetal scans, prenatal maternal trait and state anxiety were associated with increased resting state functional connectivity between sensorimotor and brainstem areas and reduced connectivity between temporoparietal cortices and basal ganglia.

**Meaning:**

Our findings suggest an association between prenatal exposure to anxiety and disrupted connectivity of neural networks.

## Teaser

This cohort study assesses the association of prenatal maternal psychological distress with in vivo fetal brain resting state functional connectivity.

## Introduction

Up to 50% of women report symptoms of stress, depression, or anxiety during pregnancy based on systematic reviews.^1,2,3^ Maternal mental health disorders are associated with adverse pregnancy outcomes and an increased risk for neuropsychiatric disorders, such as autism and attention-deficit/hyperactivity disorder.^4,5,6,7^ The high prevalence of prenatal psychological distress and its association with poor obstetric outcomes as well as motor deficits, sociocognitive, and socioaffective impairments in exposed children underscores the need for identifying the earliest effects of in utero exposure to the developing brain.

Available clinical and imaging evidence supports the negative association of maternal stress with postnatal growth and brain development. Previous studies have reported low birth weights^8,9^ in neonates exposed to high antenatal stress or anxiety. Clinical studies have reported neurobehavioral deficits beginning in infancy and early childhood, including higher reactivity,^10^ impaired motor coordination,^11^ and language delays.^12^ Postnatal brain imaging findings have provided insights into potential neural substrates for these deficits. These include reduced cortical thickness,^13,14^ amygdala and hippocampal volume changes,^15,16,17^ asymmetric electroencephalographic patterns in the frontal lobes,^18,19^ white matter microstructural changes,^20,21^ and impaired connectivity.^22,23^ More recently, a study by Wu and colleagues^24^ provided, to our knowledge, the first report of impaired brain metabolism, reduced hippocampal growth, and accelerated cortical folding in fetuses of women experiencing psychological distress. However, the effect of prenatal maternal stress on the developing neural circuitry during this critical period of brain development has not been investigated.

In this study, we investigated the association between maternal psychological distress and in vivo resting state brain functional connectivity in late second- to third-trimester fetuses. We hypothesized that elevated maternal psychological distress would be associated with disturbances in functional connectivity in neural circuitry related to stress, anxiety, or depression. Herein, psychological distress refers to symptoms of prenatal maternal depression, stress, or anxiety that have not been clinically diagnosed as a mental health illness or disorder.^24,25^ Using multivariate distance matrix regression (MDMR), we examined the association between maternal psychological distress using well-validated self-report questionnaires and the developing connections in the human fetal brain. Our goal was to assess the association between in utero exposure to elevated levels of stress, depression, and anxiety and the fetal connectome that may serve as an early biomarker of altered brain development and later neurodevelopmental disabilities.

## Methods

### Participants

Healthy pregnant women with normal ultrasonography and fetal biometry findings were prospectively recruited as part of a cohort study to investigate fetal brain development in complex congenital heart disease. Fetuses with known or suspected genetic or chromosomal abnormalities and fetuses of pregnant women with known psychiatric, metabolic, or genetic disorders; complicated pregnancies (ie, preeclampsia and gestational diabetes); multiple pregnancies; alcohol and tobacco use; maternal medications; and contraindications to magnetic resonance imaging (MRI) were excluded. Of the women who were scanned and who answered the questionnaires, only those with resting state data that met the criteria described below were included in the analysis. The institutional review board of Children’s National in Washington DC, approved this study. Written informed consent was obtained from each study participant. This study followed the Strengthening the Reporting of Observational Studies in Epidemiology (STROBE) reporting guideline.

### Maternal Stress, Anxiety, and Depression Scores

Widely used and validated stress, anxiety, and depression questionnaires were administered to participants on the same day they had a fetal MRI. These were the Perceived Stress Scale [PSS],^26^ Edinburgh Postnatal Depression Scale [EDPS],^27^ Spielberger State Anxiety Inventory [SSAI], and Spielberger Trait Anxiety Inventory [STAI].^28^ The PSS is a 10-item questionnaire that evaluates an individual’s perceived stress level over the past month. The EPDS has a similar number of items and assesses depression over the past week. Both the STAI and SSAI are composed of 20 items and evaluate trait (ie, general feeling) and state (ie, feeling for the day) anxiety, respectively. Scores of 15 or higher, 10 or higher, and 40 or higher in the PSS, EPDS, and SSAI and STAI indicate that the individual has symptoms of stress, depression, and anxiety, respectively. Scoring above these thresholds does not mean a clinical diagnosis of stress or anxiety; instead, these cutoffs are used to identify individuals who may need additional intervention.

### MRI Acquisition

A 1.5 Tesla MRI scanner (GE Healthcare) with an 8-channel receiver coil was used to acquire images of the fetal brain. Anatomical T2- weighted images (ie, sagittal, axial, and coronal sections) were collected using a single-shot fast spin-echo sequence with the following settings: TR, 1100 ms; TE, 160 ms; flip angle, 90°; and section thickness, 2 mm. Resting state echo planar images (EPI) were collected using the following parameters: TR, 3000 ms; TE, 60 ms; voxel size, 2.578 × 2.578 × 3 mm; flip angle, 90°; field of view, 33 cm; matrix size, 128 × 128; and scan duration, 7 minutes.

### Processing of Resting State Data

Fetal resting state data were preprocessed using tools from the Analysis of Functional NeuroImages unless otherwise indicated.^29^ Fetal EPI images based on blood oxygen level dependent (BOLD) contrast were slice-time corrected, followed by exclusion of the first 4 volumes of the time series, and then manually oriented to radiologic convention using an age-matched gestational age template.^30^ Resting state data were then despiked, bias-field corrected (using the N4BiasFieldCorrection tool^31^), and corrected for motion.^32,33^ After motion correction, EPI images were manually aligned to the T2-weighted brain images to ensure overlap between EPI and anatomic images; this improved later automatic affine coregistration. The EPI images were then intensity scaled to a global mode of 1000^34^ and smoothed using an isotropic 5-mm full width at half maximum gaussian kernel. After smoothing, bandpass filtering (0.009-0.08 Hz), nuisance regression with volume censoring, and normalization to a 32-week gestational age template were performed.^35,36,37^ Residual BOLD signals were analyzed.

Regressors included tissue- and motion-based signals.^35,38,39^ Specifically, tissue signals were derived from white matter and cerebrospinal fluid defined using an in-house deep learning–based segmentation algorithm^40^ and registered onto EPI images. Motion regressors included linearly detrended rigid motion parameters, their temporal derivatives, and quadratic terms.^41,42^ To further minimize the effects of motion on functional connectivity, high motion volumes, defined as those with frame-to-frame translational motion greater than 1 mm and rotational motion less than 1.5°, were censored from the hemodynamic time series.^37,43,44,45,46^ The preceding frame was also removed. Volumes in which more than 10% of voxels had signals deviating from the voxel time series’ median absolute deviation were excluded. Only fetuses with 4 or more minutes of available data after processing were included in the analyses.

### Statistical Analysis

The BOLD signals were measured from 100 regions of interest (ROIs) defined using a spectral clustering algorithm applied on functional data^47^ and refined using intensity-based masking.^48,49^ The location of the 100 ROIs are shown in the eFigure in the Supplement. Functional ROIs were named based on their overlap with the newborn automated anatomical labeling template (eTable 3 in the Supplement lists all ROI labels); as such, labels are estimated locations in the brain and do not refer to precise anatomic locations. Whole-brain differences in connectivity across all 100 ROIs associated with maternal depression, anxiety, and stress scores were then evaluated using MDMR.^50,51^ Functional connectivity across all ROI pairs (4950 connections) was computed at the subject level using pairwise Pearson correlations. These scores were *z* transformed to facilitate statistical analysis. Differences in connectivity profiles for each ROI among all participants were then evaluated using a Manhattan distance, a metric commonly used for high-dimensional data.^52^ These steps yielded an *n* × *n* distance matrix, where *n* is the number of participants, for each ROI; this distance matrix represents the dissimilarity among individual fetal connectivity networks. An MDMR was then performed^51^ to test how maternal neurobehavioral test scores accounted for differences in connectivity profiles.^53,54^ The significance of the MDMR pseudo–*F* statistic was assessed using permutation testing (permutations, 100 000). From the factors that we initially evaluated (gestational age at the time of the scan, head motion [ie, mean framewise displacement], SSAI score, STAI score, PSS, and EPDS score), we only included those that helped explain variability in connectivity profiles. These regressors included remaining volumes after preprocessing, SSAI scores, and STAI scores. The omnibus MDMR model and main effects were considered significant at a 2-tailed *P* < .05.

We then used enrichment analysis to characterize the significant main associations in the MDMR model. Enrichment analysis is widely used in large-scale genomic studies.^55^ Recently, it has been used for analyzing associations between behavior and functional connectivity.^37,56^ We first subdivided the 100 ROIs into groups using a community detection algorithm that was set up to detect smaller module sizes.^57^ Modules are nonoverlapping ROI clusters that tend to connect densely with other members of its subgroup and sparsely to the rest of the network. We then used enrichment analysis to identify within- and between-module connections in which significant associations between maternal neurobehavioral scores and functional connectivity (or, resting state functional connectivity [RSFC]) were clustered. We computed the Pearson correlation between maternal scores and all 4950 ROI-ROI pairwise connections. We then used a 1-*df* χ^2^ test to assess whether the actual number of strong brain-behavior associations (*P <* .05) within a functional network pair was more than what would be expected if all strong RSFC-behavior associations were equally distributed across all possible network pairs (ie, enrichment). The significance of the χ^2^ test was assessed by comparing the statistic with values generated using permutation testing, by which connectivity was correlated with 1000 permuted values of the maternal questionnaire score. Interactions between network modules were visualized using the BrainNet Viewer.^58^

## Results

This analysis included 59 resting-state MRI scans performed on 50 fetuses between 24.71 and 39.43 gestational weeks (mean [SD] gestational age, 33.52 [4.00] weeks). Of these fetuses, 26 (44.1%) were female and 24 (40.7%) were male. All fetuses had structurally normal brains on conventional MRIs that were evaluated by an experienced pediatric neuroradiologist (G.V.). The median Apgar score at 5 minutes for the fetuses was 9 (range, 6-10); the mean (SD) birth weight was 3308.51 (511.37) g. The mean (SD) age of the pregnant women scanned was 33.77 (5.51) years. Most of the pregnant women in the study were college graduates (43 [86%]) and reported professional employment (41 [82%]). Table 1 gives a summary of the cohort.

**Table 1.** Maternal and Fetal Clinical Characteristics

Characteristic	Individuals
**Fetal**	
Gestational age at time of scan, mean (SD), wk	33.52 (4)
Sex, No. (%)	
Female	26 (44.1)
Male	24 (40.7)
**Birth measures**	
APGAR 5 score, median (range)	9 (6-10)
Gestational age at birth, mean (SD), wk	39.07 (1.51)
Birth weight, mean (SD), g	3308.51 (511.37)
Head circumference, mean (SD), cm	34.43 (1.55)
Delivery type, No. (%)	
Spontaneous	30 (59)
Induced	16 (32)
Delivery method, No. (%)	
Vaginal	33 (66)
Caesarian	15 (30)
**Maternal**	
Age, mean (SD), y	33.77 (5.51)
Educational level, No (%)	
High school	3 (6)
Partial college	3 (6)
College graduate	16 (32)
Graduate degree	27 (54)
Employment, No. (%)	
Professional	41 (82)
Skilled, clerical, or sales	2 (4)
Unemployed or homemaker	6 (12)

### Quality Assurance of Resting State Data

The mean (SD) scan duration for fetuses was 5.4 (0.87) minutes (range, 4-7 minutes; equivalent to a mean [SD] of 108.7 [17.3] remaining brain volumes) (eTable 1 in the Supplement), and the mean (SD) maximum framewise displacement was 1.34 (0.18) mm. eTable 2 in the Supplement gives additional details on head motion.

### Maternal Stress, Depression, and Anxiety Scores

Of the 50 pregnant women, 4 (8%) were positive for state anxiety, 6 (12%) for trait anxiety, 7 (14%) for stress, and 1 (2%) for depression. Mean (SD) scores on the questionnaires were as follows: SSAI, 26.66 (6.72) (range, 20-48); STAI, 28.09 (6.62) (range, 20-50); PSS, 9.27 (5.13) (range, 1-25); and EPDS, 3.24 (2.84) (range, 0-14).

### Modules in the Fetal Brain

The fetal functional network was decomposed into 14 modules. The majority of modules were confined to 1 hemisphere except for a few, such as module 2, which included bilateral superior frontal and anterior cingulate gyri. The regions that comprised each module are shown in the Figure, A.

**Figure.**
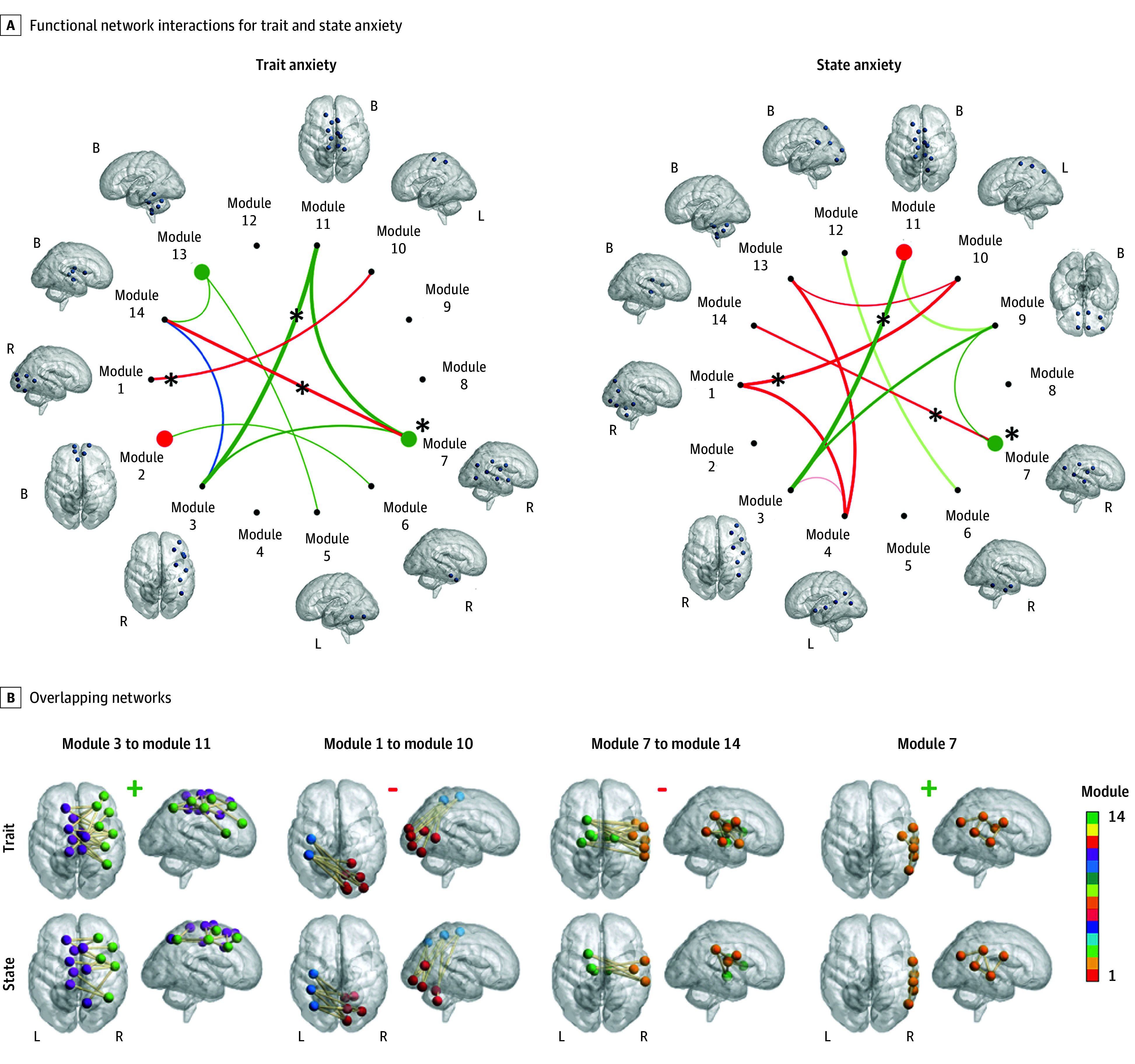
Associations Between Resting State Functional Connectivity and Behavior in the Fetal Brain Fetal blood oxygen level dependent signals were measured from 100 regions of interest (ROIs); estimated locations on the fetal brain surface are shown in the eFigure in the Supplement. A, ROIs were grouped into 14 modules. Blue spheres in brains are the locations of participating ROIs per module. Red lines indicate negative ROI-ROI correlations; green lines, positive correlations; and blue lines, 50% positive. Thicker lines indicate more ROI-ROI connections per module pair. Asterisks indicate network interactions that overlap for trait and state anxiety. B, Significant individual connections between modules pairs are shown as well as whether associations with behavior were positive (+) or negative (-). B indicates bilateral; L, left; and R, right.

### Association Between Psychological Distress Scores and Connectivity

In the MDMR model, the association between psychological distress and connectivity was significant (pseudo-*R*^2^ = 0.056, *P =* .03). The association between STAI score and connectivity was significant (pseudo-*R*^2^ = 0.021, *P* = .007), as was SSAI (pseudo-*R*^2^ = 0.019, *P* = .04) (Table 2). For the STAI, there were 285 connections with significant connectivity associated with maternal trait anxiety scores. Of these 4950 connections, 140 (2.8%) connections were positively correlated with trait anxiety and 145 (2.9%) were negatively correlated with STAI scores. For the SSAI, there were 235 strong association between behavior and RSFC. Of these, 96 were positively associated and 139 negatively correlated with maternal SSAI scores. eTables 4 and 5 in the Supplement list the ROI-ROI pairs that were associated with trait and state anxiety, respectively.

**Table 2.** Multivariate Distance Matrix Regression of Whole-Brain Connections

Factor	Statistic	*R* ^2^	*P* value
Omnibus	0.059	0.056	.03
Residual volumes	0.020	0.018	.16
SSAI score	0.022	0.019	.04
STAI score	0.024	0.021	.007

Abbreviations: SSAI, Spielberger State Anxiety Inventory; STAI, Spielberger Trait Anxiety Inventory.

### Networks Associated With Prenatal Maternal Anxiety

Enrichment analysis showed that significant associations between STAI and brain connectivity tended to cluster across 12 functional network pairs involving 8 of the 14 modules (Figure, B). Three of these were within-module (modules 2, 7, and 13) interactions; the rest were between modules. Of the 285 strong associations between RSFC and STAI, 100 (35%) connected these functional networks; 73 (73%) of these connections showed a positive association with trait anxiety.

Figure, B shows the 13 significantly enriched functional network pairs for SSAI; the majority of these were between-network links. Of the 235 significant brain-behavior interactions, 88 were clustered across 13 network pairs formed by 11 modules. Connectivity between most (58%) of the ROI-ROI pairs were inversely associated with maternal state anxiety scores.

Three network-pairs were involved in both state and trait anxiety. These pairs were modules 1 and 10, modules 7 and 14, and modules 3 and 11. Most of the ROI-ROI pairs in the first 2 network pairs were negatively associated with STAI and SSAI. Module 10 included areas of the left inferior parietal lobule and middle frontal gyrus, and module 1 comprised occipital regions. The deep gray matter and midbrain were components of module 14, and the areas surrounding the supramarginal gyrus were part of module 7. Modules 3 and 11 comprised bilateral mid-superior frontal gyrus and sensorimotor regions. Table 3 and Table 4 show the top 10 ROI-ROI connections that were associated with maternal trait and state anxiety.

**Table 3.** Strongest Positive and Negative Correlations Between Resting State Functional Connectivity and STAI Score

Rank	ROI 1	ROI 2	*r*	*P* value
**Positive correlations**				
1	PoCG-R	MCG-L	0.40	.002
2	PoCG-R	SFGdor-L	0.40	.002
3	Medulla-L	CRB-L	0.39	.002
4	Medulla-L	Midbrain-R	0.38	.003
5	Medulla-L	Pons-L	0.37	.004
6	SFGdor-R	SFGdor-R	0.37	.003
7	SFGdor-R	MTG-R	0.37	.003
8	FFG-L	Medulla-L	0.36	.005
9	PoCG-R	SPG-R	0.36	.005
10	FFG-L	Pons-R	0.36	.005
**Negative correlations**				
1	HES-R	PAL-L	–0.41	.001
2	MOG-R	PreCG-L	–0.37	.004
3	TPOsup-R	PAL-L	–0.35	.006
4	TPOsup-R	THA-L	–0.35	.007
5	MTG-R	PAL-L	–0.34	.008
6	SFGdor-R	THA-L	–0.34	.008
7	MTG-R	THA-L	–0.34	.009
8	LING-R	IPL-L	–0.34	.009
9	TPOsup-R	THA-R	–0.33	.01
10	SMG-R	PAL-L	–0.33	.01

Abbreviations: HES, Heschl gyrus; IPL, inferior parietal lobule; L, left; LING, lingual gyrus; MOG, middle occipital gyrus; MTG, middle temporal gyrus; PAL, pallidum; PreCG, precentral gyrus; R, right; ROI, region of interest; SFGdor, superior frontal gyrus (dorsal); SMG, supra marginal gyrus; STAI, Spielberger Trait Anxiety Inventory; THA, thalamus; TPOsup, temporal pole (superior).

**Table 4.** Strongest Positive and Negative Correlations Between Resting State Functional Connectivity and SSAI Score

Rank	ROI 1	ROI 2	*r*	*P* value
**Positive correlations**				
1	HIP-R	CUN-L	.37	.004
2	PoCG-R	ORBinf-R	.36	.005
3	IFGoperc-R	REC-L	.36	.005
4	PoCG-R	SFGdor-L	.35	.007
5	PoCG-R	SFGdor-L	.33	.01
6	PoCG-R	MCG-L	.32	.01
7	HIP-R	MCG-R	.32	.01
8	MTG-R	REC-L	.31	.01
9	PoCG-R	ORBmid-R	.31	.02
10	PoCG-R	SFGdor-R	.30	.02
**Negative correlations**				
1	MCG-R	PCUN-R	–.43	<.001
2	LING-R	IPL-L	–.39	.002
3	MTG-L	Pons-R	–.37	.004
4	CRB-R	ANG-L	–.37	.004
5	MTG-L	Medulla-R	–.36	.005
6	PCUN-R	MCG-L	–.35	.006
7	HES-R	PAL-L	–.34	.009
8	INS-L	PCUN-R	–.33	.01
9	INS-L	PCUN-L	–.33	.01
10	SPG-R	MCG-L	–.33	.01

Abbreviations: ANG, angular gyrus; CRB, cerebellum; CUN, cuneus; HES, Heschl gyrus; HIP, hippocampus; IFGoperc, inferior frontal gyrus (opercular); INS, insula; IPL, inferior parietal gyrus; L, left; LING, lingual gyrus; MCG, middle cingulate gyrus; MTG, middle temporal gyrus; ORBinf, orbitofrontal cortex (inferior); ORBmid, orbitofrontal cortex (middle); PAL, pallidum; PCUN, precuneus; PoCG, posterior central gyrus; R, right; REC, rectus gyrus; ROI, region of interest; SFGdor, superior frontal gyrus (dorsal); SPG, superior parietal gyrus; SSAI, Spielberger State Anxiety Inventory.

## Discussion

We report for the first time, to our knowledge, alterations in fetal functional connectivity associated with maternal anxiety. Connectivity strength between some regions correlated positively with maternal anxiety as measured using the STAI and SSAI and negatively in others. These associations between anxiety and RSFC were observed in multiple functional networks. Involved networks for state and trait anxiety overlapped; for instance, connectivity between the inferior parietal lobule and contralateral occipital regions was negatively associated with both trait and state anxiety. Likewise, links to the superior dorsal-frontal areas of the brain, mainly the somatosensory areas, were positively associated with both types. In some cases, the associations between state and trait anxiety and RSFC implicated distinct functional networks; for instance, brainstem and fusiform face area associations were positively associated with symptoms of trait anxiety only.

Our study revealed large-scale brain networks that were affected by increasing levels of anxiety in pregnant women. Although the neurobiological mechanisms underlying the effects of maternal anxiety on fetal brain development have yet to be fully explored, neurobehavioral and brain imaging studies^59,60,61,62,63,64^ have reported an association of maternal anxiety with atypical brain development. Fetal ultrasonography studies during the third trimester, for instance, have shown that fetuses of anxious women demonstrated increased wakefulness and heart rate variability,^59,60,61^ a pattern that may be related to the disorganized sleep-wake cycles observed in various neuropsychopathologies in children and adults.^62^ Numerous postnatal neurobehavioral studies^63,64^ have also shown an association between prenatal maternal anxiety and cognitive and emotional development of children.

The current literature^59,65^ suggests that cortisol mediates some of these outcomes. Fetal cortisol levels have been found to linearly correlate with maternal anxiety levels,^65^ and excess amounts in the fetus may disrupt the development of the hypothalamic-pituitary-axis, limbic system, and prefrontal areas.^59^ Mineralocorticoid and glucocorticoid receptors are ubiquitous in the brain,^66^ and this may explain the wide array of adverse neurodevelopmental outcomes reported in children exposed to prenatal anxiety. Rather than localized effects, anxiety likely impacts multiple neural systems. The involvement of different networks in our study is consistent with this. Epigenetic processes also appear to be involved. Maternal anxiety has been associated with both decreased DNA methylation at cytosine-phosphate-guanine (CpG) sites in the brain^67^ and increased DNA methylation in the placenta.^68^ Similar to cortisol receptors, there are numerous CpG islands (ie, areas of the genome with a high frequency of a consecutive cytosine and guanine nucleotides) in the brain, and their methylation has been implicated in embryonic and adult neurogenesis.^69^ Abnormal methylation has also been linked to neurologic deficits (ie, neural tube defects).^70^ We speculate that the prevalence of CpG sites in the brain,^67^ as in the case of cortisol receptors, may account for some of the distributed associations of maternal anxiety with fetal functional connectivity.

Mood disorders have been shown to disrupt large scale network organization.^71,72^ Altered interactions between the salience network (ie, brainstem, fronto-insular and dorsal anterior cingulate cortices),^73,74^ default mode network (ie, posterior cingulate, precuneus and medial prefrontal cortices),^75,76^ and executive control networks (ie, dorsolateral prefrontal and lateral and posterior parietal cortices)^77^ have been demonstrated in anxiety.^78,79^ Of interest, our findings showed that some of the more consistently affected regions and connections in the fetal connectome belonged to these networks. For example, we showed strengthened associations between areas of the brain involved with arousal and salience, such as connections between the brainstem and sensorimotor and dorsal frontal regions and anterior cingulate with the hippocampus, with increasing levels of maternal trait anxiety. Also, various brain nuclei related to anxiety reside in the brainstem, possibly helping mediate the observed increase in connectivity. Connections between other areas associated with stress and anxiety, such as the hippocampus and the insula, also showed increased connectivity associated with increased symptoms of maternal anxiety.

Although hippocampal connectivity was positively associated with trait and state anxiety scores, the hippocampal circuits activated varied. In trait anxiety, the right hippocampus interacted with the medial and dorsal superior frontal gyrus; in state anxiety, the connections were mostly to the precuneus and middle cingulate region. These connectivity profiles have previously been described in high anxiety states in adults,^80^ but the specificity of each network to either state or trait anxiety is unclear. Previous studies^81,82^ have also suggested that anterior and posterior hippocampal connectivity differs, with the former engaging with the ventromedial prefrontal cortex and likely responsible for moment-to-moment or state changes. Although our findings suggest changes specific to type of anxiety, namely state and trait, additional studies that focus on fetal hippocampal connectivity with a larger sample of pregnant women exhibiting symptoms of either trait or state anxiety will help to further elucidate selective vulnerability of specific brain regions. Longitudinal studies that allow assessment of the severity and chronicity of anxiety would be especially insightful. Studies like these are important given the altered hippocampal growth trajectories and altered connectivity in infants^15^ and children^83^ exposed to prenatal anxiety.

Our data showed an association between reduced connectivity in regions that are part of the executive control network and increasing maternal anxiety. The frontoparietal cortices were some of the more commonly affected areas with reduced connectivity. For example, the strength of connectivity between the inferior parietal lobule and superior to middle frontal cortex decreased in association with increasing levels of maternal anxiety. The association between inferior parietal lobule-occipital and fusiform connectivity and increased maternal state and trait anxiety was also observed; the same neurocircuitry has been implicated in social anxiety in adults.^84^ Default mode network–related regions, such as the medial frontal cortex and angular gyrus, also showed an association of reduced connectivity with increased maternal anxiety.

Our study is, to our knowledge, the first to report an association between maternal trait and state anxiety and altered fetal functional brain connectivity, supporting a fetal programming hypothesis. This builds on findings from previous studies in newborns, infants, children, and adults exposed to anxiety in utero that have shown network dysfunction, including reduced amygdala-thalamus connectivity,^22^ lack of inferior frontal cortex modulation in an endogenous cognitive control task,^85^ and aberrant amygdala and prefrontal cortex circuitry.^86^ Our resting state findings are also consistent with volumetric and diffusion tensor imaging studies that showed structural abnormalities in the limbic, temporal, and frontal regions in fetuses and infants exposed to prenatal distress.^87^ Hippocampal volumes have been shown to be reduced in fetuses^24^ of women with elevated stress levels. Similarly, slower hippocampal growth has been reported in infants of women with anxiety symptoms, suggesting an association of prenatal maternal anxiety with regional brain growth.^15^ In newborns and infants exposed to anxiety in utero, fractional anisotropy, a diffusion tensor imaging metric that reflects neuronal integrity, has been shown to be decreased in regions critical to emotional and cognitive development (the insula and dorsolateral frontal regions), visual processing (middle occipital cortex), and social functioning (angular region and posterior cingulate),^88^ areas also affected in the current study. Taken together, these findings suggest that prenatal maternal anxiety may affect the development of a diverse set of brain regions and networks, including temporal and frontal and prefrontal areas, and that this may, in turn, impact long-term neurodevelopmental outcomes.

In our study, we showed that functional connections between areas that developmentally associate earlier (ie, brainstem and sensorimotor areas and local short-range connections) were stronger in high maternal trait anxiety states. In contrast, those that emerged later in development (ie, more distant anteroposterior, interhemispheric connections) were weakened by higher levels of anxiety.^89,90,91^ Strengthening of earlier emerging connections suggests a preference for networks that support more fundamental processes (ie, sensory and motor processing, arousal) as opposed to connections between association regions that will eventually subserve higher-order cognitive functions such as executive control. This may reflect an adaptive response of the fetal brain to in utero exposure to anxiety, the mechanisms of which require further investigation that is outside the scope of the current study.

### Limitations

This study has limitations. We examined a moderately large sample of fetuses, but younger fetuses (ie, at lower gestational ages) were not equally represented in the sample owing in part to the technical difficulties associated with the acquisition and processing of these images. As a result, investigating the onset and timing of connectivity changes remained challenging. We anticipate that improvements in acquisition and processing techniques will help alleviate some of these issues. A larger sample that also includes clinically diagnosed pregnant women, may also give better power to detect associations between other types of maternal psychological distress (ie, stress and depression) and functional brain connectivity. Second, only 12% of the women in the study were positive for trait anxiety using the STAI, and only 8% presented with symptoms of state anxiety using the SSAI. A complete understanding of the functional connectome in fetuses exposed to prenatal anxiety entails an assessment of women with higher anxiety scores and clinically diagnosed and managed anxiety disorders. However, our study emphasizes the need for mental health surveillance in pregnant women as functional brain changes appears at subclinical levels. Third, maternal anxiety was measured using self-report. Although biases may be inherent to this method, well-validated and tested questionnaires have been shown to reliably quantify subjective perceptions of psychologic distress.^92^ To complement surveys, studies are under way that use objective measures of stress and anxiety. Fourth, to maximize the number of data sets analyzed, few fetuses had 2 scans included in the sample. Although this number accounts for a small subset of the total cohort, futures studies that account for repeated scans within the context of the analytic technique used (ie, MDMR) would be ideal. Fifth, whether the functional connectivity findings observed during the fetal period predict infant and childhood neurobehavioral outcomes needs to be validated. Longitudinal follow-up studies that evaluate postnatal neurodevelopmental outcomes in these fetuses are currently under way.

## Conclusions

In this cohort study, alterations in late second- to third-trimester fetal brain functional connectivity were associated with maternal anxiety. Maternal anxiety and fetal connectivity correlations either decreased or increased depending on the networks involved. Interhemispheric connections, such as those involving the medial frontal regions and basal ganglia, were found to be weakened. In contrast, connections such as those between the brainstem and sensorimotor areas, were strengthened in association with higher trait anxiety scores. Some networks were associated with both trait and state anxiety and overlapped, whereas some were distinct to 1 type. Areas associated with salience network, DMN, and central executive network were commonly implicated. These findings suggest an association between altered fetal programming in fetuses and maternal anxiety and the need for mental health surveillance and interventions for pregnant women.

## Notes

Supplement. **eTable 1.** Number of frames analyzed **eTable 2.** Summary of frame-by-frame motion **eTable 3.** ROIs and ROI labels **eTable 4.** Connections significantly associated with maternal trait anxiety scores arranged by decreasing *r* values (positive to negative correlations) **eTable 5.** Connections significantly associated with maternal state anxiety scores arranged by decreasing *r* values (positive to negative correlations) **eFigure.** 100 Regions of interest projected on the surface of a 32-week fetal brain
