# Supplementary material for: Association of Prenatal Maternal Anxiety With Fetal Regional Brain Connectivity
Source: JAMA Netw Open. 2020 Dec 7;3(12):e2022349. doi: 10.1001/jamanetworkopen.2020.22349 (PMC12549102; doi:10.1001/jamanetworkopen.2020.22349)
Supplement: Supplement. — eTable 1. Number of frames analyzed eTable 2. Summary of frame-by-frame motion eTable 3. ROIs and ROI labels eTable 4. Connections significantly associated with maternal trait anxiety scores arranged by decreasing r values (positive to negative correlations) eTable 5. Connections significantly associated with maternal state anxiety scores arranged by decreasing r values (positive to negative correlations) eFigure. 100 Regions of interest projected on the surface of a 32-week fetal brain [file jamanetwopen-e2022349-s001.pdf]

Supplemental Online Content

De Asis-Cruz J, Krishnamurthy D, Zhao L, et al. Association of prenatal maternal anxiety with fetal regional brain connectivity. *JAMA Netw Open*. 2020;3(12):e2022349. doi:10.1001/jamanetworkopen.2020.22349

eTable 1. Number of frames analyzed
eTable 2. Summary of frame-by-frame motion
eTable 3. ROIs and ROI labels
eTable 4. Connections significantly associated with maternal trait anxiety scores arranged by decreasing *r* values (positive to negative correlations)
eTable 5. Connections significantly associated with maternal state anxiety scores arranged by decreasing *r* values (positive to negative correlations)
eFigure. 100 Regions of interest projected on the surface of a 32-week fetal brain

This supplemental material has been provided by the authors to give readers additional information about their work.

© 2020 De Asis-Cruz J et al. *Journal Network Open.*

eTable 1. Number of frames analyzed.

Data	Retained	% of Total		Data	Retained	% of Total		Data	Retained	% of Total
1	90	64.29		21	127	90.71		41	113	80.71
2	106	75.71		22	99	70.71		42	105	75.00
3	136	97.14		23	122	87.14		43	117	83.57
4	112	80.00		24	133	95.00		44	96	68.57
5	109	77.86		25	82	58.57		45	107	76.43
6	84	60.00		26	123	87.86		46	98	70.00
7	82	58.57		27	93	66.43		47	109	77.86
8	109	77.86		28	134	95.71		48	87	62.14
9	101	72.14		29	108	77.14		49	83	59.29
10	134	95.71		30	100	71.43		50	118	84.29
11	104	74.29		31	83	59.29		51	140	100.00
12	98	70.00		32	107	76.43		52	103	73.57
13	126	90.00		33	80	57.14		53	132	94.29
14	140	100.00		34	102	72.86		54	85	60.71
15	90	64.29		35	125	89.29		55	123	87.86
16	92	65.71		36	115	82.14		56	94	67.14
17	123	87.86		37	95	67.86		57	116	82.86
18	110	78.57		38	104	74.29		58	82	58.57
19	126	90.00		39	126	90.00		59	129	92.14
20	104	74.29		40	140	100.00				

© 2020 De Asis-Cruz J et al. *Journal Network Open.*

eTable 2. Summary of frame-by-frame motion.

Metric	Mean	STD
Ave frame by frame motion, x (mm)	0.12	0.04
Ave frame by frame motion, y (mm)	0.13	0.04
Ave frame by frame motion, z (mm)	0.16	0.05
Ave frame by frame motion, pitch (°)	0.21	0.07
Ave frame by frame motion, yaw (°)	0.21	0.07
Ave frame by frame motion, roll (°)	0.21	0.07
Max frame by frame motion, x (mm)	0.52	0.17
Max frame by frame motion, y (mm)	0.54	0.15
Max frame by frame motion, z (mm)	0.64	0.19
Max frame by frame motion, pitch (°)	1.02	0.31
Max frame by frame motion, yaw (°)	1.04	0.26
Max frame by frame motion, roll (°)	1.07	0.28
Ave framewise displacement	0.50	0.13
Max framewise displacement	1.34	0.18

© 2020 De Asis-Cruz J et al. *Journal Network Open.*

eTable 3. ROIs and ROI labels.

IDX	ROI Name	Label	IDX	ROI Name	Label
1	brainstem (midbrain)-L	BSmid-L	51	Thalamus right	THA-R
2	Inferior frontal gyrus (triangular) left	IFGtriang-L	52	Superior frontal gyrus (dorsal) right	SFGdor-R
3	Inferior parietal lobule left	IPL-L	53	Anterior cingulate gyrus right	ANG-R
4	brainstem (pons)-L	BSpons-L	54	Middle occipital gyrus right	MOG-R
5	Middle cingulate gyrus left	MCG-L	55	Superior frontal gyrus (medial) right	SFGmed-R
6	Orbitofrontal cortex (middle) left	ORBmid-L	56	Cuneus right	CUN-R
7	Precuneus left	PCUN-L	57	Cerebellum right	CRB-R
8	Cerebellum left	CRB-L	58	Postcentral gyrus right	PoCG-R
9	Superior temporal gyrus left	STG-L	59	Calcarine cortex right	CAL-R
10	Rectus gyrus left	REC-L	60	Anterior cingulate gyrus right	ACG-R
11	Inferior temporal gyrus left	ITG-L	61	Superior frontal gyrus (dorsal) right	SFGdor-R
12	Calcarine cortex left	CAL-L	62	Middle temporal gyrus right	MTG-R
13	Middle temporal gyrus left	MTG-L	63	brainstem (midbrain)-R	BSmid-R
14	Middle frontal gyrus left	MFG-L	64	Inferior frontal gyrus (opercular) right	IFGoperc-R
15	Inferior frontal gyrus (opercular) left	IFGoperc-L	65	Precuneus right	PCUN-R
16	Parahippocampal gyrus left	PHG-L	66	Lingual gyrus right	LING-R
17	Superior occipital gyrus left	SOG-L	67	Cerebellum right	CRB-R
18	Cerebellum left	CRB-L	68	Heschl gyrus right	HES-R
19	brainstem (medulla)-L	Bsmed-L	69	Inferior temporal gyrus right	ITG-R
20	Precuneus left	PCUN-L	70	Orbitofrontal cortex (superior) right	ORBsupb-R
21	Inferior temporal gyrus left	ITG-L	71	Supplementary motor area right	SMG-R
22	Anterior cingulate gyrus left	ACG-L	72	Precuneus left	PCUN-L
23	Middle frontal gyrus left	MFG-L	73	Anterior cingulate gyrus right	ACG-R
24	Middle occipital gyrus left	MOG-L	74	Putamen right	PUT-R
25	Orbitofrontal cortex (inferior) left	ORBinf-L	75	Middle frontal gyrus right	MFG-R
26	Middle cingulate gyrus right	MCG-R	76	Superior occipital gyrus right	SOG-R
27	Thalamus left	THA-L	77	brainstem (medulla)-R	BSmed-R
28	Cuneus left	CUN-L	78	Fusiform gyrus right	FFG-R
29	Middle temporal gyrus left	MTG-L	79	Supplementary motor area right	SMA-R
30	Rectus gyrus left	REC-L	80	Postcentral gyrus right	PoCG-R
31	Superior frontal gyrus (medial) left	SFGmed-L	81	Superior frontal gyrus (dorsal) right	SFGdor-R
32	Postcentral gyrus left	PoCG-L	82	Superior occipital gyrus right	SOG-R
33	Cuneus left	CUN-L	83	Fusiform gyrus right	FFG-R
34	Rolandic operculum left	ROL-L	84	Inferior parietal lobule right	IPL-R
35	Middle cingulate gyrus left	MCG-L	85	Rectus gyrus right	REC-R
36	Superior frontal gyrus (dorsal) left	SFGdor-L	86	Middle temporal gyrus right	MTG-R
37	Anterior cingulate gyrus left	ANG-L	87	Temporal pole (superior) right	TPOsup-R
38	Posterior cingulate gyrus left	PCG-L	88	brainstem (pons)-R	BSpons-R
39	Fusiform gyrus left	FFG-L	89	Superior parietal gyrus right	SPG-R
40	Superior frontal gyrus (dorsal) left	SFGdor-L	90	Middle frontal gyrus right	MFG-R
41	Precentral gyrus left	PreCG-L	91	Temporal pole (middle) right	TPOmid-R
42	Inferior parietal lobule left	IPL-R	92	Insula right	INS-R
43	Calcarine cortex left	CAL-L	93	Middle temporal gyrus right	MTG-R
44	Pallidum left	PAL-L	94	Postcentral gyrus right	PoCG-R
45	Temporal pole (superior) left	TPOsup-L	95	Middle cingulate gyrus right	MCG-R
46	Middle temporal gyrus left	MTG-L	96	Inferior frontal gyrus (opercular) right	IFGoperc-R
47	Superior frontal gyrus (medial) left	SFGmed-L	97	Orbitofrontal cortex (inferior) right	ORBinf-R
48	Inferior temporal gyrus left	ITG-L	98	Orbitofrontal cortex (middle) right	ORBmid-R
49	Thalamus left	THA-L	99	Hippocampus right	HIP-R
50	Inferior parietal lobule left	IPL-L	100	Inferior temporal gyrus right	ITG-R

© 2020 De Asis-Cruz J et al. *Journal Network Open.*

eTable 4. Connections significantly associated with maternal trait anxiety scores arranged by decreasing *r* values (positive to negative correlations).

	ROI 1	ROI 2	r	p
1	brainstem (medulla)-L	SFGdor-R	0.44	0.0006
2	REC-L	PCG-L	0.43	0.0007
3	CAL-L	brainstem (medulla)-L	0.42	0.0009
4	MCG-L	PoCG-R	0.40	0.0016
5	SFGdor-L	PoCG-R	0.40	0.0019
6	CRB-L	brainstem (medulla)-L	0.39	0.0021
7	brainstem (pons)-L	ANG-R	0.39	0.0023
8	brainstem (medulla)-L	brainstem (midbrain)-R	0.38	0.0027
9	CRB-L	PCUN-L	0.38	0.0032
10	brainstem (pons)-L	brainstem (medulla)-L	0.37	0.0035
11	SFGdor-R	SFGdor-R	0.37	0.0036
12	SFGdor-R	MTG-R	0.37	0.0036
13	CRB-L	SFGdor-R	0.37	0.0038
14	brainstem (medulla)-L	PoCG-R	0.37	0.0042
15	brainstem (medulla)-L	FFG-L	0.36	0.0048
16	PoCG-R	SPG-R	0.36	0.0052
17	FFG-L	brainstem (pons)-R	0.36	0.0053
18	LING-R	FFG-R	0.36	0.0053
19	SFGmed-L	IFGoperc-R	0.36	0.0054
20	brainstem (medulla)-L	PoCG-R	0.36	0.0055
21	SPG-R	MTG-R	0.35	0.0060
22	CAL-L	brainstem (pons)-R	0.34	0.0076
23	CRB-L	brainstem (midbrain)-R	0.34	0.0082
24	MFG-R	MTG-R	0.33	0.0097
25	SFGdor-R	brainstem (medulla)-R	0.33	0.0098
26	brainstem (medulla)-R	brainstem (pons)-R	0.33	0.0100
27	MOG-R	CUN-R	0.33	0.0101
28	PoCG-R	MCG-R	0.33	0.0103
29	SFGdor-R	TPOmid-R	0.33	0.0103
30	FFG-L	CUN-R	0.33	0.0105
31	CRB-L	brainstem (midbrain)-R	0.33	0.0107
32	THA-L	PCUN-L	0.33	0.0109
33	brainstem (pons)-L	FFG-L	0.33	0.0111
34	MTG-L	SFGdor-R	0.33	0.0117
35	SFGmed-R	PoCG-R	0.32	0.0120
36	MCG-L	IPL-R	0.32	0.0127
37	PHG-L	CUN-L	0.32	0.0132
38	SFGdor-R	PoCG-R	0.32	0.0135
39	PUT-R	ITG-R	0.32	0.0139
40	PoCG-R	MCG-R	0.32	0.0144
41	MTG-R	INS-R	0.32	0.0149
42	PoCG-R	ORBinf-R	0.32	0.0150
43	SMA-R	PoCG-R	0.31	0.0152
44	SFGmed-L	PoCG-R	0.31	0.0154
45	FFG-L	brainstem (medulla)-R	0.31	0.0162
46	ANG-R	brainstem (medulla)-R	0.31	0.0167
47	SFGdor-L	MFG-R	0.31	0.0170
48	brainstem (pons)-L	CAL-L	0.31	0.0170
49	PUT-R	IFGoperc-R	0.31	0.0177
50	SMG-R	SMA-R	0.31	0.0180
51	SFGdor-L	PoCG-R	0.31	0.0185

© 2020 De Asis-Cruz J et al. *Journal Network Open.*

52	MTG-L	MFG-R	0.31	0.0187
53	brainstem (medulla)-L	PCG-L	0.30	0.0189
54	MTG-L	ANG-R	0.30	0.0193
55	SFGdor-R	PoCG-R	0.30	0.0200
56	SFGdor-R	ORBinf-R	0.30	0.0204
57	MCG-L	MTG-R	0.30	0.0208
58	SMA-R	MTG-R	0.30	0.0209
59	PoCG-R	INS-R	0.30	0.0209
60	MTG-R	PoCG-R	0.30	0.0221
61	SFGmed-L	PoCG-R	0.30	0.0222
62	CRB-L	FFG-L	0.30	0.0226
63	brainstem (medulla)-R	PoCG-R	0.29	0.0235
64	REC-L	SFGmed-L	0.29	0.0237
65	brainstem (pons)-L	ITG-L	0.29	0.0241
66	SFGdor-L	MFG-R	0.29	0.0244
67	MTG-R	SMG-R	0.29	0.0249
68	PCUN-L	ORBsupb-R	0.29	0.0255
69	SFGdor-R	PoCG-R	0.29	0.0255
70	MCG-L	PoCG-R	0.29	0.0264
71	PoCG-R	HES-R	0.29	0.0269
72	PoCG-R	CRB-R	0.29	0.0275
73	SFGdor-L	MTG-R	0.29	0.0281
74	brainstem (medulla)-L	brainstem (pons)-R	0.29	0.0282
75	FFG-L	brainstem (midbrain)-R	0.29	0.0283
76	THA-R	PoCG-R	0.28	0.0287
77	CUN-L	ITG-L	0.28	0.0292
78	FFG-L	MOG-R	0.28	0.0292
79	brainstem (pons)-L	CUN-R	0.28	0.0302
80	MCG-R	THA-L	0.28	0.0304
81	ACG-L	IFGoperc-R	0.28	0.0305
82	MTG-L	ITG-R	0.28	0.0305
83	IPL-L	ITG-R	0.28	0.0308
84	PoCG-R	MTG-R	0.28	0.0314
85	IPL-R	ITG-R	0.28	0.0328
86	SFGmed-L	HIP-R	0.28	0.0329
87	SFGdor-R	MFG-R	0.28	0.0330
88	PoCG-L	ITG-R	0.28	0.0334
89	brainstem (pons)-L	brainstem (midbrain)-R	0.28	0.0340
90	THA-R	SMG-R	0.28	0.0340
91	CAL-R	MTG-R	0.28	0.0346
92	ORBinf-L	TPOmid-R	0.28	0.0346
93	HES-R	SMG-R	0.28	0.0347
94	INS-R	PoCG-R	0.27	0.0356
95	MTG-R	SPG-R	0.27	0.0356
96	ANG-R	IPL-R	0.27	0.0357
97	ACG-R	PoCG-R	0.27	0.0363
98	CRB-L	MOG-R	0.27	0.0364
99	SFGdor-R	SMG-R	0.27	0.0365
100	CUN-R	CRB-R	0.27	0.0371
101	IPL-R	INS-R	0.27	0.0373
102	ANG-R	CUN-R	0.27	0.0377
103	THA-R	IPL-R	0.27	0.0385
104	CUN-R	FFG-R	0.27	0.0387
105	MTG-L	PoCG-R	0.27	0.0391
106	ANG-R	MFG-R	0.27	0.0396
107	SFGdor-R	HIP-R	0.27	0.0397
108	MOG-R	CAL-R	0.27	0.0400

© 2020 De Asis-Cruz J et al. *Journal Network Open.*

109	SMG-R	ACG-R	0.27	0.0414
110	ANG-R	SMG-R	0.27	0.0416
111	SFGdor-R	brainstem (pons)-R	0.27	0.0422
112	CAL-L	brainstem (medulla)-R	0.27	0.0424
113	ACG-L	FFG-R	0.27	0.0424
114	MFG-R	HIP-R	0.27	0.0425
115	SFGdor-L	PoCG-R	0.27	0.0425
116	MCG-L	PoCG-R	0.26	0.0426
117	ACG-L	MOG-R	0.26	0.0427
118	brainstem (midbrain)-R	brainstem (medulla)-R	0.26	0.0432
119	MTG-L	SOG-R	0.26	0.0434
120	SFGdor-R	MTG-R	0.26	0.0435
121	MCG-L	MFG-R	0.26	0.0436
122	SOG-R	MFG-R	0.26	0.0437
123	SFGmed-L	SFGdor-R	0.26	0.0438
124	THA-R	PoCG-R	0.26	0.0444
125	STG-L	SFGmed-L	0.26	0.0449
126	MCG-R	HIP-R	0.26	0.0450
127	SFGdor-L	PoCG-R	0.26	0.0451
128	INS-R	HIP-R	0.26	0.0453
129	brainstem (midbrain)-R	PCUN-L	0.26	0.0454
130	PCUN-L	ITG-R	0.26	0.0471
131	SFGmed-L	MTG-R	0.26	0.0473
132	CRB-L	SFGdor-R	0.26	0.0478
133	PoCG-R	INS-R	0.26	0.0482
134	brainstem (midbrain)-R	HIP-R	0.26	0.0482
135	FFG-L	CAL-L	0.26	0.0483
136	MTG-R	MTG-R	0.26	0.0486
137	FFG-R	SOG-R	0.26	0.0491
138	SFGdor-L	IPL-R	0.26	0.0492
139	brainstem (pons)-L	PCG-L	0.26	0.0495
140	MCG-L	PoCG-R	0.26	0.0497
141	IFGtriang-L	CRB-R	-0.26	0.0498
142	SOG-L	SFGdor-R	-0.26	0.0497
143	PCUN-L	MTG-L	-0.26	0.0493
144	PCUN-L	INS-R	-0.26	0.0490
145	LING-R	IPL-R	-0.26	0.0482
146	MTG-L	brainstem (medulla)-L	-0.26	0.0481
147	CUN-L	FFG-R	-0.26	0.0481
148	MCG-R	CUN-R	-0.26	0.0480
149	REC-R	MCG-R	-0.26	0.0479
150	ACG-R	HIP-R	-0.26	0.0465
151	CRB-L	ACG-R	-0.26	0.0462
152	TPOsup-L	IFGoperc-R	-0.26	0.0461
153	CRB-L	SFGmed-R	-0.26	0.0457
154	ORBmid-L	FFG-R	-0.26	0.0451
155	MFG-L	FFG-R	-0.26	0.0449
156	IPL-R	REC-R	-0.26	0.0448
157	FFG-R	TPOmid-R	-0.26	0.0444
158	IFGoperc-L	SMG-R	-0.26	0.0439
159	PoCG-L	SMG-R	-0.26	0.0439
160	REC-L	INS-R	-0.26	0.0437
161	PAL-L	FFG-R	-0.26	0.0434
162	CUN-R	MTG-R	-0.26	0.0432
163	FFG-R	MTG-R	-0.26	0.0432
164	THA-L	IPL-R	-0.26	0.0430
165	IPL-L	SMA-R	-0.26	0.0429

© 2020 De Asis-Cruz J et al. *Journal Network Open.*

166	MFG-L	MTG-L	-0.26	0.0429
167	FFG-L	ACG-R	-0.26	0.0428
168	IFGoperc-L	ACG-L	-0.26	0.0427
169	IPL-L	MOG-R	-0.27	0.0424
170	PHG-L	ANG-L	-0.27	0.0424
171	THA-L	MFG-R	-0.27	0.0423
172	ANG-L	SFGmed-L	-0.27	0.0419
173	PHG-L	IFGoperc-R	-0.27	0.0415
174	PreCG-L	ORBinf-R	-0.27	0.0414
175	brainstem (midbrain)-L	PoCG-L	-0.27	0.0409
176	PCUN-L	SFGdor-R	-0.27	0.0407
177	SFGmed-L	SFGmed-R	-0.27	0.0405
178	brainstem (midbrain)-L	MTG-L	-0.27	0.0405
179	MTG-L	brainstem (pons)-R	-0.27	0.0399
180	ITG-L	ITG-R	-0.27	0.0394
181	CRB-L	THA-L	-0.27	0.0393
182	CRB-L	CRB-R	-0.27	0.0390
183	MFG-L	IFGoperc-R	-0.27	0.0389
184	IPL-L	MFG-L	-0.27	0.0387
185	CUN-L	ANG-L	-0.27	0.0376
186	ACG-R	TPOmid-R	-0.27	0.0374
187	ROL-L	ACG-R	-0.27	0.0366
188	PreCG-L	ORBmid-R	-0.27	0.0363
189	ACG-L	SFGmed-R	-0.27	0.0363
190	PCUN-L	CAL-L	-0.28	0.0350
191	ROL-L	SMG-R	-0.28	0.0350
192	TPOsup-L	CRB-R	-0.28	0.0349
193	CAL-L	SMG-R	-0.28	0.0348
194	SFGmed-L	SFGdor-R	-0.28	0.0346
195	IPL-L	SFGdor-L	-0.28	0.0342
196	ACG-L	INS-R	-0.28	0.0340
197	CUN-L	SFGdor-L	-0.28	0.0340
198	SFGmed-L	CRB-R	-0.28	0.0334
199	MFG-L	CAL-R	-0.28	0.0333
200	THA-L	ITG-R	-0.28	0.0328
201	SOG-L	MCG-R	-0.28	0.0326
202	SFGmed-L	brainstem (medulla)-R	-0.28	0.0316
203	MTG-L	FFG-L	-0.28	0.0305
204	STG-L	LING-R	-0.28	0.0305
205	THA-L	MTG-R	-0.28	0.0303
206	CRB-L	ACG-L	-0.28	0.0292
207	PAL-L	PoCG-R	-0.28	0.0290
208	PCUN-L	PAL-L	-0.28	0.0287
209	IPL-L	CUN-R	-0.29	0.0285
210	IPL-L	MCG-R	-0.29	0.0285
211	IPL-L	FFG-R	-0.29	0.0284
212	ROL-L	MTG-R	-0.29	0.0283
213	IFGoperc-L	IFGoperc-R	-0.29	0.0266
214	MFG-L	PAL-L	-0.29	0.0266
215	PAL-L	PoCG-R	-0.29	0.0264
216	IPL-L	TPOsup-R	-0.29	0.0263
217	CRB-L	ACG-R	-0.29	0.0261
218	SOG-L	SPG-R	-0.29	0.0252
219	brainstem (midbrain)-R	SMG-R	-0.29	0.0248
220	PCUN-L	CAL-R	-0.29	0.0245
221	MFG-L	IFGoperc-R	-0.29	0.0243
222	SOG-L	brainstem (midbrain)-R	-0.29	0.0241

© 2020 De Asis-Cruz J et al. *Journal Network Open.*

223	PreCG-L	LING-R	-0.29	0.0241
224	IPL-L	SOG-R	-0.29	0.0240
225	ROL-L	THA-R	-0.30	0.0230
226	CRB-L	ORBmid-R	-0.30	0.0230
227	CAL-L	MTG-R	-0.30	0.0224
228	SFGmed-L	SOG-R	-0.30	0.0219
229	IFGoperc-L	PCG-L	-0.30	0.0217
230	PCUN-L	MCG-R	-0.30	0.0214
231	PHG-L	PoCG-R	-0.30	0.0214
232	PCUN-L	IFGoperc-R	-0.30	0.0214
233	CRB-R	TPOmid-R	-0.30	0.0213
234	MFG-L	CRB-R	-0.30	0.0205
235	PCUN-L	ANG-L	-0.30	0.0198
236	PCUN-L	FFG-R	-0.30	0.0198
237	ANG-L	CRB-R	-0.31	0.0188
238	TPOsup-L	HES-R	-0.31	0.0186
239	PoCG-L	IFGoperc-R	-0.31	0.0181
240	IPL-L	CAL-R	-0.31	0.0177
241	ACG-R	IPL-R	-0.31	0.0174
242	SFGdor-R	PCUN-R	-0.31	0.0167
243	CUN-L	IFGoperc-R	-0.31	0.0165
244	ACG-L	CAL-R	-0.31	0.0161
245	brainstem (midbrain)-L	IPL-R	-0.31	0.0160
246	brainstem (midbrain)-L	HES-R	-0.31	0.0154
247	CRB-R	TPOsup-R	-0.32	0.0150
248	REC-L	CRB-R	-0.32	0.0132
249	THA-L	SFGdor-R	-0.32	0.0131
250	IFGoperc-L	PAL-L	-0.32	0.0127
251	CRB-L	TPOsup-R	-0.33	0.0116
252	TPOsup-L	TPOsup-R	-0.33	0.0114
253	SOG-L	PoCG-R	-0.33	0.0112
254	brainstem (midbrain)-L	PoCG-R	-0.33	0.0101
255	PAL-L	SMG-R	-0.33	0.0100
256	THA-R	TPOsup-R	-0.33	0.0099
257	CRB-R	MTG-R	-0.34	0.0093
258	MCG-L	SFGmed-L	-0.34	0.0092
259	IPL-L	LING-R	-0.34	0.0090
260	SFGmed-L	CAL-R	-0.34	0.0089
261	THA-L	MTG-R	-0.34	0.0089
262	THA-L	SFGdor-R	-0.34	0.0084
263	PAL-L	MTG-R	-0.34	0.0082
264	PAL-L	SPG-R	-0.34	0.0080
265	IFGoperc-L	MFG-R	-0.34	0.0077
266	THA-L	TPOsup-R	-0.35	0.0073
267	MCG-L	PCUN-R	-0.35	0.0072
268	SFGmed-L	INS-R	-0.35	0.0071
269	PAL-L	SFGdor-R	-0.35	0.0060
270	PAL-L	TPOsup-R	-0.35	0.0059
271	SFGmed-L	MCG-R	-0.37	0.0043
272	PCUN-R	MCG-R	-0.37	0.0037
273	PreCG-L	MOG-R	-0.37	0.0035
274	IPL-R	IPL-L	-0.38	0.0034
275	MTG-L	IPL-L	-0.38	0.0033
276	PreCG-L	IFGoperc-R	-0.38	0.0029
277	MFG-L	MOG-R	-0.39	0.0024
278	PCUN-R	INS-R	-0.39	0.0023
279	PHG-L	IFGoperc-R	-0.39	0.0023

© 2020 De Asis-Cruz J et al. *Journal Network Open.*

280	ORBmid-L	CRB-R	-0.40	0.0016
281	PAL-L	HES-R	-0.41	0.0014
282	PreCG-L	SMG-R	-0.41	0.0012
283	SFGmed-L	CUN-R	-0.42	0.0010
284	MFG-L	FFG-R	-0.44	0.0005
285	ROL-L	HES-R	-0.45	0.0003

eTable 5. Connections significantly associated with maternal state anxiety scores arranged by decreasing *r* values (positive to negative correlations).

	ROI1	ROI2	r	p
1	MCG-L	PoCG-R	0.49	0.0001
2	SFGdor-L	PoCG-R	0.43	0.0006
3	SFGdor-L	PoCG-R	0.42	0.0010
4	SFGmed-R	PoCG-R	0.41	0.0012
5	SMA-R	PoCG-R	0.41	0.0014
6	MTG-L	SOG-L	0.38	0.0027
7	CUN-L	HIP-R	0.37	0.0036
8	REC-L	PCG-L	0.37	0.0037
9	SFGmed-L	PoCG-R	0.37	0.0041
10	LING-R	FFG-R	0.37	0.0042
11	brainstem (medulla)-L	SFGdor-R	0.36	0.0045
12	PoCG-R	ORBinf-R	0.36	0.0045
13	REC-L	IFGoperc-R	0.36	0.0046
14	PoCG-R	TPOmid-R	0.36	0.0048
15	PUT-R	IFGoperc-R	0.36	0.0049
16	CAL-L	brainstem (medulla)-L	0.36	0.0050
17	CAL-L	brainstem (medulla)-R	0.36	0.0056
18	SFGmed-L	IFGoperc-R	0.35	0.0063
19	SFGdor-L	PoCG-R	0.35	0.0070
20	ACG-R	PoCG-R	0.35	0.0073
21	PoCG-R	PUT-R	0.34	0.0081
22	SFGdor-L	PoCG-R	0.33	0.0098
23	PoCG-L	ACG-R	0.33	0.0118
24	MCG-L	PoCG-R	0.32	0.0132
25	IFGoperc-R	PoCG-R	0.32	0.0145
26	MCG-L	MFG-L	0.32	0.0146
27	MFG-L	ACG-R	0.32	0.0147
28	MCG-R	HIP-R	0.32	0.0149
29	REC-L	MTG-R	0.31	0.0154
30	ITG-L	PoCG-R	0.31	0.0158
31	MFG-R	TPOmid-R	0.31	0.0159
32	ORBmid-L	PoCG-R	0.31	0.0162
33	SFGdor-R	MTG-R	0.31	0.0166
34	PoCG-R	ORBmid-R	0.31	0.0171
35	TPOsup-L	MTG-L	0.31	0.0175
36	brainstem (midbrain)-L	FFG-L	0.31	0.0178
37	SOG-R	IFGoperc-R	0.31	0.0185
38	PCUN-L	ORBsupb-R	0.30	0.0190
39	SFGdor-R	PoCG-R	0.30	0.0193
40	SFGdor-R	SFGdor-R	0.30	0.0199
41	MTG-R	ORBsupb-R	0.30	0.0204

© 2020 De Asis-Cruz J et al. *Journal Network Open.*

42	MCG-L	MFG-R	0.30	0.0206
43	REC-L	MTG-R	0.30	0.0213
44	PCUN-L	IPL-R	0.30	0.0220
45	PreCG-L	ACG-R	0.30	0.0225
46	IPL-R	ORBinf-R	0.30	0.0225
47	SFGdor-R	TPOmid-R	0.30	0.0231
48	SFGdor-R	HIP-R	0.29	0.0235
49	ANG-R	IFGoperc-R	0.29	0.0249
50	MTG-R	SPG-R	0.29	0.0254
51	MFG-R	MTG-R	0.29	0.0255
52	CRB-R	ITG-R	0.29	0.0258
53	SFGdor-L	MFG-R	0.29	0.0259
54	ACG-L	PreCG-L	0.29	0.0260
55	SFGmed-L	PoCG-R	0.29	0.0261
56	ORBsupb-R	IFGoperc-R	0.29	0.0265
57	ITG-L	MTG-R	0.29	0.0269
58	SFGdor-R	brainstem (medulla)-R	0.29	0.0271
59	brainstem (pons)-L	PCG-L	0.29	0.0273
60	THA-L	IFGoperc-R	0.29	0.0281
61	MFG-R	ORBinf-R	0.28	0.0289
62	PreCG-L	REC-R	0.28	0.0292
63	PCUN-L	ITG-R	0.28	0.0296
64	PCUN-L	ORBsupb-R	0.28	0.0302
65	TPOsup-L	SOG-R	0.28	0.0307
66	MCG-R	PoCG-L	0.28	0.0311
67	ROL-L	MCG-R	0.28	0.0319
68	MTG-R	MTG-R	0.28	0.0325
69	SFGdor-L	MTG-R	0.28	0.0328
70	PoCG-R	MCG-R	0.28	0.0336
71	SFGdor-L	REC-R	0.28	0.0338
72	MTG-L	MFG-R	0.28	0.0341
73	SFGdor-R	TPOmid-R	0.28	0.0342
74	MCG-L	IPL-R	0.28	0.0343
75	SFGdor-R	ORBinf-R	0.28	0.0349
76	ITG-L	MOG-R	0.28	0.0350
77	CUN-R	CRB-R	0.27	0.0375
78	MTG-L	PoCG-R	0.27	0.0376
79	ORBinf-L	MCG-R	0.27	0.0378
80	MTG-R	SMG-R	0.27	0.0383
81	IPL-R	ITG-R	0.27	0.0388
82	PoCG-R	ORBsupb-R	0.27	0.0399
83	REC-L	PCUN-L	0.27	0.0421
84	CRB-R	ITG-R	0.26	0.0425
85	SMG-R	MTG-R	0.26	0.0429
86	PCUN-R	ORBmid-R	0.26	0.0441
87	FFG-L	brainstem (medulla)-R	0.26	0.0444
88	PoCG-R	PCUN-R	0.26	0.0447
89	PreCG-L	MTG-R	0.26	0.0450
90	brainstem (medulla)-L	PoCG-R	0.26	0.0453
91	ITG-L	MTG-R	0.26	0.0467
92	MFG-R	HIP-R	0.26	0.0470
93	PoCG-R	MTG-R	0.26	0.0488
94	PCUN-L	HIP-R	0.26	0.0492
95	THA-L	PoCG-R	0.26	0.0495
96	ANG-R	SMG-R	0.26	0.0497
97	ROL-L	PCG-L	-0.26	0.0500
98	FFG-R	MFG-R	-0.26	0.0487

© 2020 De Asis-Cruz J et al. *Journal Network Open.*

99	REC-R	MCG-R	-0.26	0.0485
100	CAL-L	SMG-R	-0.26	0.0482
101	IPL-L	CRB-L	-0.26	0.0480
102	PCUN-L	PCUN-L	-0.26	0.0477
103	MTG-L	brainstem (medulla)-R	-0.26	0.0472
104	ACG-L	TPOsup-L	-0.26	0.0472
105	CRB-L	ANG-L	-0.26	0.0471
106	brainstem (midbrain)-L	HES-R	-0.26	0.0470
107	PAL-L	LING-R	-0.26	0.0469
108	PHG-L	REC-R	-0.26	0.0466
109	THA-L	HES-R	-0.26	0.0466
110	REC-L	MCG-R	-0.26	0.0452
111	TPOsup-L	brainstem (medulla)-R	-0.26	0.0451
112	IPL-L	FFG-R	-0.26	0.0449
113	TPOsup-L	CRB-R	-0.26	0.0445
114	REC-L	HIP-R	-0.26	0.0445
115	MTG-L	PoCG-R	-0.26	0.0436
116	PUT-R	HIP-R	-0.26	0.0434
117	CUN-L	IPL-R	-0.26	0.0428
118	PreCG-L	PoCG-R	-0.26	0.0427
119	CUN-L	ITG-R	-0.27	0.0421
120	PAL-L	PUT-R	-0.27	0.0421
121	PreCG-L	LING-R	-0.27	0.0420
122	MTG-L	CRB-R	-0.27	0.0417
123	SFGdor-L	SPG-R	-0.27	0.0417
124	PoCG-L	ORBinf-R	-0.27	0.0410
125	ACG-R	IPL-R	-0.27	0.0402
126	PAL-L	SFGdor-R	-0.27	0.0399
127	IPL-L	LING-R	-0.27	0.0398
128	FFG-R	TPOmid-R	-0.27	0.0396
129	CRB-L	THA-L	-0.27	0.0396
130	PCUN-L	SFGdor-R	-0.27	0.0394
131	SMA-R	IFGoperc-R	-0.27	0.0394
132	THA-R	ORBmid-R	-0.27	0.0387
133	CRB-L	PreCG-L	-0.27	0.0386
134	IPL-L	CUN-R	-0.27	0.0384
135	ITG-L	REC-R	-0.27	0.0377
136	TPOsup-L	HES-R	-0.27	0.0376
137	REC-L	CAL-L	-0.27	0.0362
138	PAL-L	SMG-R	-0.28	0.0349
139	IFGtriang-L	PCG-L	-0.28	0.0348
140	ORBinf-L	ITG-R	-0.28	0.0344
141	MTG-L	CRB-R	-0.28	0.0342
142	PCUN-L	MCG-R	-0.28	0.0339
143	SPG-R	INS-R	-0.28	0.0337
144	SOG-L	FFG-R	-0.28	0.0335
145	PCG-L	TPOsup-R	-0.28	0.0334
146	IFGoperc-L	PCG-L	-0.28	0.0333
147	MTG-R	brainstem (pons)-R	-0.28	0.0333
148	PreCG-L	CRB-R	-0.28	0.0332
149	SMA-R	SPG-R	-0.28	0.0330
150	ORBinf-L	IPL-L	-0.28	0.0326
151	MTG-L	CRB-R	-0.28	0.0324
152	SFGdor-L	MCG-R	-0.28	0.0322
153	ACG-L	MCG-R	-0.28	0.0317
154	CUN-L	brainstem (midbrain)-R	-0.28	0.0315
155	MFG-L	FFG-R	-0.28	0.0314

© 2020 De Asis-Cruz J et al. *Journal Network Open.*

156	FFG-R	MCG-R	-0.28	0.0313
157	MCG-L	FFG-L	-0.28	0.0299
158	CUN-L	FFG-R	-0.28	0.0295
159	ITG-L	ITG-R	-0.28	0.0295
160	IPL-L	FFG-R	-0.28	0.0293
161	CRB-L	TPOsup-L	-0.28	0.0287
162	MTG-L	brainstem (medulla)-R	-0.29	0.0283
163	CUN-L	PUT-R	-0.29	0.0283
164	PreCG-L	IFGoperc-R	-0.29	0.0273
165	MTG-L	brainstem (medulla)-L	-0.29	0.0270
166	PCUN-L	PCUN-L	-0.29	0.0269
167	FFG-R	INS-R	-0.29	0.0266
168	CAL-L	THA-R	-0.29	0.0266
169	MTG-L	IPL-L	-0.29	0.0265
170	CUN-L	ANG-L	-0.29	0.0263
171	STG-L	MTG-R	-0.29	0.0262
172	MTG-L	PHG-L	-0.29	0.0260
173	MTG-L	CRB-R	-0.29	0.0255
174	SFGmed-L	INS-R	-0.29	0.0250
175	SFGmed-L	CAL-R	-0.29	0.0243
176	REC-L	REC-R	-0.29	0.0243
177	MFG-L	MOG-R	-0.29	0.0237
178	SFGmed-L	CRB-R	-0.29	0.0234
179	TPOsup-L	FFG-R	-0.30	0.0233
180	PCUN-L	FFG-R	-0.30	0.0232
181	ROL-L	SFGmed-L	-0.30	0.0229
182	IPL-L	PCUN-L	-0.30	0.0229
183	CRB-R	HES-R	-0.30	0.0223
184	REC-L	IFGoperc-L	-0.30	0.0221
185	ROL-L	ACG-R	-0.30	0.0219
186	MTG-L	PoCG-R	-0.30	0.0211
187	MCG-L	FFG-R	-0.30	0.0208
188	PCUN-L	LING-R	-0.30	0.0207
189	SPG-R	MCG-R	-0.30	0.0202
190	brainstem (midbrain)-R	SMG-R	-0.30	0.0199
191	brainstem (pons)-L	PreCG-L	-0.30	0.0192
192	PHG-L	IFGoperc-R	-0.31	0.0185
193	CRB-L	SOG-R	-0.31	0.0183
194	MCG-L	PCG-L	-0.31	0.0178
195	PHG-L	ANG-L	-0.31	0.0175
196	PCUN-L	CAL-R	-0.31	0.0164
197	CRB-R	TPOsup-R	-0.31	0.0161
198	STG-L	HES-R	-0.31	0.0157
199	CRB-L	CRB-R	-0.31	0.0156
200	CRB-R	SPG-R	-0.31	0.0153
201	REC-L	INS-R	-0.31	0.0153
202	PCUN-L	FFG-R	-0.32	0.0139
203	IPL-L	CRB-R	-0.32	0.0137
204	CAL-L	ITG-R	-0.32	0.0127
205	IFGoperc-R	brainstem (pons)-R	-0.32	0.0124
206	MCG-L	SPG-R	-0.33	0.0120
207	PCUN-L	CAL-R	-0.33	0.0118
208	PCUN-L	INS-R	-0.33	0.0110
209	PCUN-R	INS-R	-0.33	0.0106
210	SFGmed-L	MCG-R	-0.33	0.0105
211	PreCG-L	MTG-L	-0.33	0.0102
212	REC-L	ROL-L	-0.33	0.0097

© 2020 De Asis-Cruz J et al. *Journal Network Open.*

213	ROL-L	SMG-R	-0.34	0.0095
214	PAL-L	HES-R	-0.34	0.0092
215	CRB-L	TPOsup-R	-0.34	0.0091
216	ROL-L	HES-R	-0.34	0.0078
217	CAL-L	MTG-R	-0.34	0.0078
218	CRB-L	ORBmid-R	-0.35	0.0070
219	CAL-L	ANG-R	-0.35	0.0063
220	MCG-L	PCUN-R	-0.35	0.0061
221	MTG-L	brainstem (medulla)-R	-0.36	0.0052
222	PHG-L	PoCG-L	-0.36	0.0046
223	REC-L	ROL-L	-0.36	0.0045
224	IFGoperc-L	SFGmed-L	-0.37	0.0040
225	ANG-L	CRB-R	-0.37	0.0040
226	CAL-L	MFG-R	-0.37	0.0039
227	MTG-L	brainstem (pons)-R	-0.37	0.0037
228	ROL-L	MTG-R	-0.37	0.0037
229	CRB-L	IFGoperc-R	-0.38	0.0032
230	ROL-L	MTG-R	-0.38	0.0027
231	ROL-L	MTG-R	-0.39	0.0025
232	IPL-L	LING-R	-0.39	0.0020
233	PCUN-R	MCG-R	-0.43	0.0008
234	LING-R	IPL-R	-0.47	0.0002
235	PreCG-L	SMG-R	-0.48	0.0001

© 2020 De Asis-Cruz J et al. *Journal Network Open.*

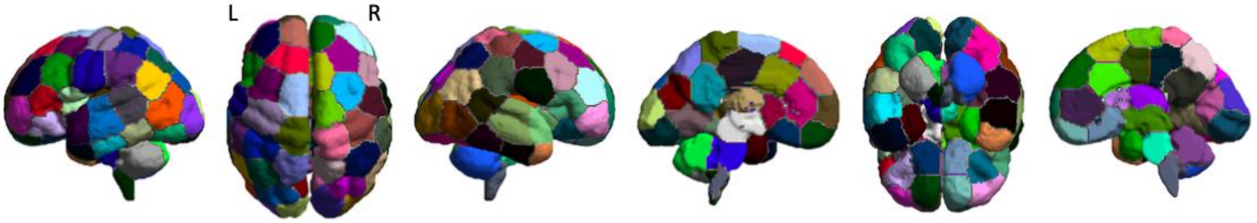

eFigure1. 100 regions of interest projected on the surface of a 32-week fetal brain. L – left, R – right.

© 2020 De Asis-Cruz J et al. *Journal Network Open.*
